# Antibiotics in Canadian poultry productions and anticipated alternatives

**DOI:** 10.3389/fmicb.2014.00282

**Published:** 2014-06-17

**Authors:** Moussa S. Diarra, François Malouin

**Affiliations:** ^1^Pacific Agri-Food Research Center, Agriculture and Agri-Food CanadaAgassiz, BC, Canada; ^2^Département de Biologie, Faculté des Sciences, Centre d'Étude et de Valorisation de la Diversité Microbienne, Université de SherbrookeSherbrooke, QC, Canada

**Keywords:** growth promoters, non-therapeutic antibiotics, alternatives to antibiotics, cranberry, c-di-GMP, poultry production, broilers

## Abstract

The use of antibiotics in food-producing animals has significantly increased animal health by lowering mortality and the incidence of diseases. Antibiotics also have largely contributed to increase productivity of farms. However, antibiotic usage in general and relevance of non-therapeutic antibiotics (growth promoters) in feed need to be reevaluated especially because bacterial pathogens of humans and animals have developed and shared a variety of antibiotic resistance mechanisms that can easily be spread within microbial communities. In Canada, poultry production involves more than 2600 regulated chicken producers who have access to several antibiotics approved as feed additives for poultry. Feed recipes and mixtures vary greatly geographically and from one farm to another, making links between use of a specific antibiotic feed additive and production yields or selection of specific antibiotic-resistant bacteria difficult to establish. Many on-farm studies have revealed the widespread presence of antibiotic-resistant bacteria in broiler chickens. While some reports linked the presence of antibiotic-resistant organisms to the use of feed supplemented with antibiotics, no recent studies could clearly demonstrate the benefit of antimicrobial growth promoters on performance and production yields. With modern biosecurity and hygienic practices, there is a genuine concern that intensive utilization of antibiotics or use of antimicrobial growth promoters in feed might no longer be useful. Public pressure and concerns about food and environmental safety (antibiotic residues, antibiotic-resistant pathogens) have driven researchers to actively look for alternatives to antibiotics. Some of the alternatives include pre- and probiotics, organic acids and essential oils. We will describe here the properties of some bioactive molecules, like those found in cranberry, which have shown interesting polyvalent antibacterial and immuno-stimulatory activities.

## Introduction

Since the discovery of penicillin by Fleming in 1928, several antibiotics which can be classified based on their molecular targets in bacteria (cell wall, protein synthesis, nucleic acids, folic acid metabolism) have been marketed for the treatment of infectious diseases both in animals and humans. The agents used in the treatment of animals and humans often belong to the same classes of antibiotics having similar modes of action and bacterial cell targets. This interface brings a variety of problems and worries. Bacteria developing resistance to these drugs in animals may be transmitted to humans or spread their mechanisms of resistance, which may eventually be found in human pathogens. Such a situation may lead to the loss of therapeutic efficacy in both veterinary and human medicine.

It is evident that antibiotics substantially improved public health. For example, since their discovery about 70 years ago, antibiotics have greatly reduced mortality and morbidity associated with infectious diseases and have increased life expectancy around the world. In addition to their therapeutic use, antibiotics also are deployed in animals for prophylaxis and growth promotion (improvement of animal zootechnical parameters). For example, antibiotics such as ceftiofur (a third generation cephalosporin), bacitracin (polypeptide) and virginiamycin (streptogramin) are used in poultry production to respectively prevent and control infections (respiratory diseases and necrotic enteritis) and to improve food conversion and body-weight gain. The use of antibiotics as growth promoters was adopted in the 1940s when animals fed dried mycelia of *Streptomyces aureofaciens* containing chlortetracycline residues showed improved performances (Castanon, 2007). It has been estimated that antibiotic growth promoters in animals, through unspecific and not well defined mechanisms, improve bodyweight by 5–6% and feed efficiency by 3–4%, with the most pronounced effects observed in young animals (Butaye et al., 2003). However, the deployment of antimicrobial agents can change the bacterial environment by eliminating susceptible strains, and only allowing antibiotic resistant bacteria (i.e., those with higher fitness) to survive (O'Brien, 2002). Antimicrobial agents may thus modify the intestinal microflora and create a favorable environment for establishment of resistant and pathogenic bacteria. Accordingly, positive associations were found between the presence of certain virulence genes and antibiotic resistance determinants (Aslam et al., 2012; Johnson et al., 2012). The impact of antimicrobial growth promoters on the development of antimicrobial resistant bacteria has been the subject of several reports and led to their ban in the European Union in 2006.

The poultry industry has grown and improved in recent years due to the continuous integration of various disciplines for production such as poultry health, nutrition, breeding, husbandry, and knowledge of poultry products (Anonymous, 2007). For example, in 1928, the average broiler required 112 days and 22 kg of feed to reach 1.7 kg. Since 1990, broilers required about 35–42 days and 4 kg of feed to reach 2 kg (National Research Council, 1999). Even though this improvement could be attributable in part to antibiotics, relevance of their use as growth promoters in feed needs to be re-evaluated. With modern broiler production practices, a broiler body weight of 1.8 kg can be reached by using 3.2 kg of feed in 35 days without addition of any antibiotic in feed (Diarra et al., 2007). In this chapter, we will review the use of antimicrobial agents in the Canadian poultry industry and discuss public health issues and concerns related to antibiotic resistant bacteria. We also will explore possible alternatives that could be developed in respect to food and environmental safety as well as to public and animal health and welfare.

## Antibiotic selective pressure

The use of antibiotics as growth promoters is negatively perceived because pathogenic bacteria of humans and animals have developed and shared a variety of antibiotic resistance mechanisms that can be easily spread within microbial communities. Nowadays, worldwide spread of antibiotic resistance mechanisms resulting from selective pressures (use of antibiotics) has undeniably reduced treatment options and therapeutic efficacy in human medicine. However, the relative responsibility of selective pressures occasioned by human medicine, veterinary or agricultural practices is still unclear. Furthermore, metagenomic studies have established some links between resistance mechanisms found in microorganisms from the environment and the clinic (Perry and Wright, 2013), making even more difficult the identification of the primary cause of selective pressure and support arguments for multiple sources of antibiotic resistance genes (Lupo et al., 2012).

Transformation and conjugation are mechanisms accommodating gene transfer among bacteria and are believed to play important roles in the rapid spread of antibiotic resistance (Chen et al., 2005). In addition, the horizontal transfer of mobile genetic elements also contributes to the evolution of emerging pathogens through dissemination of virulence genes. A variety of genetic materials, such as plasmids, can participate to this evolution (Carattoli, 2013). Moreover, integrative and conjugative elements (ICEs) can be disseminated through transferable elements like conjugative plasmids but can also integrate into the genome of new bacterial hosts (Burrus and Waldor, 2004). Transposons are also other mobile genetic elements that can contain antibiotic resistance gene cassettes such as resistance integrons (Hall, 2012). class 1 integrons, which can be disseminated through a wide variety of taxonomically divergent bacteria, are often found in bacteria associated with livestock and poultry (Mathew et al., 2007). Another mean for gene transfer across bacterial species of different taxa includes transduction (gene transfer mediated by bacteriophages) as evidenced by using a metagenomic approach for antibiotic resistance genes (Muniesa et al., 2013). Noteworthy, antibiotic resistance gene transfer can be insidious as phenotypic detection of inducible antibiotic resistance may be difficult and may account for the “silent” spread of such genes in bacterial communities (Chancey et al., 2012).

Hence, some bacterial isolates of animal origin might not be pathogenic to humans but they may carry and disseminate important antibiotic resistance genes. For example, the same *vanA* gene cluster involved in vancomycin resistance could be detected in enterococci of both human and animal origins, indicating horizontal transfer of gene clusters between enterococci of different origins (Conly, 2002; Hammerum, 2012). Similarly, multidrug-resistant commensal *E. coli* of animal origin represent an important reservoir of antibiotic resistance genes that can be transferred to other strains and bacterial species through contact with other animals or humans and through contaminated food (Szmolka and Nagy, 2013). Many food animals are now broadly recognized as carriers of livestock-associated pathogens that can in many occurrences cause diseases in the human host. For example, Livestock-Associated Methicillin-Resistant *Staphylococcus aureus* (LA-MRSA) have been transmitted from cows or pigs to humans and could cause diseases (Witte et al., 2007; Garcia-Alvarez et al., 2011; Laurent et al., 2012). Also recently, it was suggested that multiple cases of community-acquired urinary tract infections (UTI) caused by antibiotic-resistant bacteria could be considered outbreaks of foodborne origins (Nordstrom et al., 2013). In Canada, studies suggested that poultry meats could play a role in human infections (Manges et al., 2007) and that chicken represented the most probable reservoir of extraintestinal pathogenic *E. coli* causing UTI (Bergeron et al., 2012). Certainly, in view of the complexity of the antibiotic resistance spread allowed by various means (genes, resistant commensals, or resistant pathogens) from various reservoirs (food and environment), global coordinated actions are required (Marshall and Levy, 2011; Laxminarayan et al., 2013). Toward a global action in the Canadian poultry industry, at least two reasonable questions should arise. Are antibiotics acting as growth promoters still needed nowadays? What are possible alternatives to antibiotics that could be used in preserving poultry health while maintaining farm profitability, food safety and environmental health?

## Poultry industry in canada

According to the Food and Agriculture Organization (FAO) of the United Nations, the world chicken production was estimated at 71,851,372 tons in 2005, up 3% from the previous year (Lacobucci et al., 2006). It is interesting to note that chicken production has been grown steadily worldwide since the early 1990s. From 1985 to 2005, 158% growth was recorded. The leading chicken-producing countries include the United States, China, the European Union and Brazil. In 2005, those four countries or group of countries accounted for about 61% of world chicken production. Canada was the thirteenth-largest chicken-producing country in 2005 with 981.2 million kilograms representing 1.4% of the world's production (Lacobucci et al., 2006).

Poultry production is an important industry in Canada. In 2012, the value of Canadian chicken products was estimated at $2.4 billion, involving 2645 regulated chicken producers and a large number of businesses associated with chicken farming http://www.agr.gc.ca/poultry/index_eng.htm). In the same year, Canada produced 1.02 billion kilograms of chicken (eviscerated weight), 60% of which was produced in the provinces of Quebec and Ontario. Canadian domestic consumption was 30 kilograms per person and retail purchases accounted for approximately 634 million kilograms representing 62% of Canada's total consumption. Canada exported over 5.9 million chicks to 13 countries; a commercial value estimated at over $14.5 million in 2012. The United States was the largest market (91%). Other countries included Mexico, Japan, the Philippines, and China (http://www.agr.gc.ca/poultry/index_eng.htm).

In Canada, several medical ingredients are approved as feed additives for poultry farmers. Among them, several classes of antimicrobial agents, such as glycolipids (bambermycin), polypeptides (bacitracin), ionophores (salinomycin), β-lactams (penicillin), streptogramins (virginiamycin), and tetracyclines (chlortetracycline) are used in broiler production for growth promotion and prevention of infectious diseases (Table 1). For broilers, salinomycin and bacitracin are widely used in starter, grower or finisher feeds while virginiamycin is used in the finisher. Many of the above antimicrobials are effective against Gram-positive bacteria such as *Clostridium perfringens*, the etiological cause of necrotic enteritis which is one of the main disease concerns for poultry producers worldwide (Stutz et al., 1983; Heredia and Labbé, 2001; Shojadoost et al., 2013). Since *C. perfringens* also is one of the foodborne pathogens associated with poultry, it is believed that antimicrobial agents targeting this pathogen also help to prevent any potential food safety problems.

**Table 1:** **Agents approved as medicating ingredients in Canadian poultry feed**.

**Agents**	**Chicken**	**Turkey**
Arsanilic acid	GP	GP
Bacitracin (zinc or methylene disalicylate)	GP, NE, EM	GP
Zinc bacitracin and procaine penicillin	EM	
Bambermycin	GP	GP
Chlortetracycline hydrochloride	GP, OT, ST	CRD, GP, HE, NE, OT, spE, ST
Oxytetracycline hydrochloride	OT, ST	CRD, OT, spE, ST, SI, SY
Virginiamycin	GP, NE	
3-nitro-4-hydroxyphenylarsonic acid	GP	GP
Penicillin procaine	GP	
Amprolium	CO	CO
Clopidol	CO	
Decoquinate	CO	
Diclazuril	CO	CO
Halofuginone hydrobromide	CO	
Lasalocid sodium	CO	CO
Maduramicin ammonium	CO	CO
Monensin sodium	CO	CO
Narasin	CO, NE	
Narasin and nicarbazin	CO	
Nicarbazin	CO	
Robenidine hydrochloride	CO	CO
Salinomycin sodium	CO	
Semduramicin sodium	CO	
Zoalene	CO	CO
Hygromycin B	WO	
Piperazine	WO	WO
Tylosin phosphate	NE	
Nitarsone (4-Nitrophenylarsonic acid)		BH
Novobiocin		SY

Abbreviations: BH, blackhead; CO, coccidiosis; CRD, chronic respiratory disease; EM, early mortality; GP, growth promotion and/or feed efficiency; HE, hexamitiasis; NE, necrotic enteritis; OT, other nutritional uses; spE, non-specific enteritis, ST, stress; SY, synovitis; SI, sinusitis; WO, worms (Government of Canada, 2013a).

The preventive use of antibiotics in poultry production may impact therapeutic efficacy in human medicine. Ceftiofur is a third-generation cephalosporin, marketed for use in turkey, cattle, swine, lambs, dogs, and horses. This antibiotic is often subcutaneously injected in day old chicks (0.17 mg/chick) or into eggs (0.08–0.20 mg) as a prophylactic measure in Canada to prevent the yolk-sac infection (omphalitis), a costly disease caused by *Escherichia coli* (Canadian Medical Association, 2009). This use is under Canadian provincial regulations which differ from province to province (Government of Canada, 2002). Overall data on antibiotic use in Canadian hatcheries are not available; however, it seems that about 30% of the chicks hatched in the province of Ontario would be treated mostly with ceftiofur followed by gentamicin (Rosengren et al., 2009). Ceftiofur is not used in humans, however, its analog ceftriaxone, another third-generation cephalosporin, is an important medical antibiotic used in humans. Resistance to these related antibiotics can be mediated by similar mechanisms involving genes such as *bla*_CMY−2_, an AmpC-type β-lactamase that hydrolyzes third-generation cephalosporins.

In Canada as well as in several countries, various combinations of antimicrobial agents are used in feed depending on birds' ages, formulation and mixtures and such recipes greatly vary geographically and from one farm to another. Hence, despite the intuitive link between antibiotic usage in poultry and the emergence of antibiotic resistant bacteria, variations in antimicrobial usage make links between the use of specific feed additives and the selection of specific antibiotic resistant bacteria difficult to establish (Diarrassouba et al., 2007). Furthermore, the origins of antibiotic resistant bacteria remain uncertain and the sources are certainly numerous (Marshall and Levy, 2011). Consequently, antibiotic resistance in commensal enterococci can be found as early as in 1-day old chicks (Table 2).

**Table 2:** **Antibiotic susceptibility phenotypes of some enterococci isolates from day-old chicks before placement and of some enterococci isolates found in freshly manufactured feed (starter, grower, and finisher)**.

		**Antibiotic susceptibility phenotype^b^**									
**Category^a^**	**Antibiotic**	**Day-old chick isolates**							**Feed isolates**		
		**1**	**2**	**3**	**4**	**5**	**6**	**7**	**Starter**	**Grower**	**Finisher**
I	Ciprofloxacin	R	I	S	S	S	S	S	I	S	I
	Daptomycin	S	S	S	S	S	S	S	S	S	S
	Linezolid	S	S	S	S	S	S	S	S	S	S
	Vancomycin	S	I	S	S	I	S	I	S	S	S
II	Erythromycin	R	S	R	R	I	R	S	S	S	I
	Gentamicin	S	S	S	S	S	S	S	S	S	S
	Kanamycin	R	S	R	R	S	R	S	S	S	S
	Lincomycin	R	R	R	R	R	R	R	R	R	R
	Penicillin	S	S	S	S	S	S	S	S	S	S
	Q/D^c^	R	S	R	R	S	S	S	R	S	S
	Streptomycin	R	S	R	R	S	R	S	S	S	S
	Tylosin	R	S	R	R	S	R	S	S	S	S
III	Chloramphenicol	S	S	S	S	S	S	S	S	S	S
	Nitrofurantoin	S	S	S	S	S	S	S	I	S	S
	Tetracycline	R	R	R	R	R	R	R	S	S	R
	Bacitrcin	R	R	R	R	R	R	R	R	R	R
IV	Flavomycin	R	S	R	R	R	R	S	R	R	R

a Category indicates antibiotic ranking based on importance in human medicine.

b The antibiotic susceptibility phenotypes are presented as S, sensitive; R, resistant; I, intermediary, using CIPARS susceptibility breakpoints (Government of Canada, 2013b).

c Quinupristin/Dalfopristin.

### Growth promoters and performance

Few studies have been performed to demonstrate the economic benefits of antimicrobial growth promoters in the Canadian poultry production system (Table 3). In controlled studies, the effects of diet supplementation with bambermycin, penicillin, salinomycin, bacitracin, salinomycin-bacitracin, virginiamycin, chlortetracycline, monensin, and narasin on body weight, feed intake, feed efficiency, and mortality were evaluated (Diarra et al., 2007; Bonnet et al., 2009). No significant difference was noted between the treatment groups for the overall performance although virginiamycin and penicillin improved feed efficiency. The experiment conducted by Dumonceaux et al. (2006) found that dietary inclusion of virginiamycin increased body weight and improved feed efficiency from days 0 to 15 but that no difference was noted for bird's performance parameters for the remainder of the study. The used of chlortetracycline as a feed supplement at a rate permitted in Canada has been reported to induce no significant improvement in 21- and 42-day old live body weights or feed conversion efficiencies (Proudfoot et al., 1988). Avoparcin an analog of vancomycin, has not been approved in Canada, however, the growth promotion effect of this agent was reported in experimental turkeys by a Canadian study (Leeson and Summers, 1981). The economic effect of removing antibiotics used for growth promotion in commercial broiler chickens was evaluated in a non-randomized study in the USA (Graham et al., 2007). Positive production changes were associated with the use of antibiotic agents, but these benefits were insufficient to offset their cost (Graham et al., 2007). Well-designed on-farm studies should be encouraged in the Canadian poultry production system to support or not the use of growth promoting antimicrobial agents. With the improved hygienic and biosecurity practices currently observed in modern poultry production, there is a genuine concern that utilization of antibiotics as growth promoters in feed might no longer be useful.

**Table 3:** **Canadian studies evaluating growth promotion gains and health parameters of in-feed antibiotic supplementations**.

**References**	**Promoter**	**Route**	**Study objectives**	**Conclusions/Observations**
Leeson and Summers, 1981	Avoparcin (10 ppm) and robenz (33 ppm) alone or in combination	In-feed: Turkeys	Performance and carcass grades	Avoparcin improved weight gain irrespective of coccidiostat robenz inclusion. Feed utilization and carcass grades were not influenced by diet
Proudfoot et al., 1988	Chlortetracycline (5.5 mg/kg)	In-feed: Broiler	Growth promotion	No further gain
Proudfoot et al., 1990	Lincomycin (2.2 ppm)	In-feed or in water: Broiler	Growth promotion	No effect on mortality, efficiency of food utilization, final body weights or monetary indices
Dumonceaux et al., 2006	Virginiamycin (20 ppm)	In-feed: Broiler	Performance; intestinal microbiota	Improved body weight and feed efficiency from 0 to 15 days. Increased abundance *Lactobacillus* spp. in the proximal digestive tract with fewer targets affected in the distal regions
Guban et al., 2006	Bacitracin and monensin alone or in combination (0.5 g/kg)	In-feed: Broiler	Growth performance; population levels of *Lactobacillus salivarius*; bile salts deconjugation	Bacitracin increased feed intake and decreased conversion ratio while improving weight gain and concentrations of conjugated bile salts. Monensin increased fat digestibility. Antimicrobials reduced populations of *Lactobacillus salivarius*
Diarra et al., 2007	Bambermycin (2 ppm), penicillin (2.2 ppm), salinomycin (60 ppm), and bacitracin (55 ppm) or a combination of salinomycin (60 ppm) + bacitracin (ppm)	In-feed: Broiler	Growth performances; pathogen counts; resistance phenotypes; resistance determinants	Except for penicillin (improvement of feed efficiency), no significant effect on performance; no effect on bacterial count in the intestine, ceca or litter. Significant effect on antimicrobial resistance phenotypes and genotypes
Brisbin et al., 2008	Virginiamycin (11 or 22 ppm)	In-feed: Broiler	Antibody response	Enhancing systemic antibody responses to some antigens
Gong et al., 2008	Bacitracin (50 ppm)	In-feed: Broiler	Ileum and caeca microbiota	Alteration of the microbiota composition in 3-day-old chicks but no effect on the microbial richness
Bonnet et al., 2009	Bambermycin (2 ppm); penicillin (2.2 ppm); salinomycin (60 ppm); and bacitracin (55 ppm); a combination of salinomycin (60 ppm) + bacitracin (ppm); chlortetracycline (110 ppm), virginiamycin (11 or 22 ppm); monensin (99 ppm); narasin (70 ppm)	In-feed: Broiler	*E. coli* pathotypes and phylogenetic group	Affect the phylogenetic group and pathotypes distribution in the gut
Baurhoo et al., 2009	Mannanoligosaccharide (0.2 or 0.5%); Virginiamycin (16.5 ppm); Bacitracin (55 ppm)	In-feed: Broiler	Performance; intestinal development; cecal and litter microbial populations; carcass parameters	No effect of antimicrobial on performance and carcass. Some effect on cecal and litter microbial population on day 34
Salim et al., 2013	Direct-fed microbial (DFM) such as *Lactobacillus reuteri* (0.1%) or a mixture of *L. reuteri, Bacillus subtilis*, and *Saccharomyces cerevisiae* (0.1%); Virginiamycin (0.1%)	In-feed: Broiler	Performance; immune response; cecal microbial population; ileal morphology	Increase performance from 0 to 21 days. DFM increases white blood cells, monocytes and the plasma immunoglobulin concentrations while decreases cecal *E. coli* population

### Growth promoters and gut microflora

The lives of human beings, livestock and poultry are closely associated with microorganisms and the microbiota of their gut plays an important role in their overall health, productivity and well-being (Callaway et al., 2008; Ley et al., 2008). The growth of normal intestinal bacteria varies with the gut environment, and there is an increasing interest in the commensal components of the gut microflora associated with food-producing animals (Yost et al., 2011). Due to public and possible food safety and environmental health concerns, the monitoring of the changes in the microbiome (microbial genomes) as a function of chicken production practices is imperative. Knowledge of the impacts of antimicrobial agents on the gut microbiome might lead to production practices that improve broiler intestinal health and growth performances.

The use of virginiamycin as a growth promoter was associated with an increased abundance of bacteria in the duodenal loop to proximal ileum, with fewer bacteria affected in the distal regions (ileocecal junction and cecum) indicating that virginiamycin modifies the composition of the chicken intestinal microbiota (Dumonceaux et al., 2006). Using the 16S rRNA gene-based polymerase chain reaction followed by denaturing gradient gel electrophoresis profiling, dietary treatment with bacitracin (50 mg/kg) has been shown to alter the composition of the microbiota but did not change its richness (Gong et al., 2008). The authors demonstrated that the impact of bacitracin was particularly obvious in 3-day-old chicks. Lactobacilli were abundant in the cecal microbiota of 3-day-old chicks regardless of the dietary treatment with bacitracin (Gong et al., 2008). Recently, metagenomic sequencing approaches demonstrated that salinomycin-feeding (60 ppm) has a profound impact on the dynamics of the chicken ceca microbiome (Fung et al., 2013). These authors showed that the salinomycin fed group had an increased abundance of the Elusimicrobia, and a decreased abundance of Chloroflexi, Cyanobacteria, and Synergistetes. For example, the abundance of *Bifidiobacterium* spp. and *Lactobacillus* spp. increased significantly in the salinomycin-fed birds compared to the untreated control group. A functional analysis of environmental gene tags (EGTs) revealed that in the salinomycin-treated birds there was an increased abundance of the cell wall and capsule, iron acquisition, motility and β-lactamase gene categories while a decrease of multidrug efflux pump EGTs was detected (Fung et al., 2013). In addition to such Canadian studies, other authors demonstrated the impact of antimicrobial growth promoters on the chicken gut microflora (Knarreborg et al., 2002; Torok et al., 2011; Singh et al., 2013). For example, pyrosequencing followed by phylogenetic analyses indicated that diet supplementation with penicillin resulted in an elevated proportion of bacteria of the phylum Firmicutes from 58.1 to 91.5% and a decreased proportion of members of the phylum Bacteroidetes from 31.1 to 2.9% in the gut microflora of broilers compared to that observed in broilers fed with the control non-supplemented diet (Singh et al., 2013). Besides, the decrease of broiler ileal sucrase and maltase activities and increase of ileal mucosal immunoglobulin A (IgA) as well as the increase of *Lactobacillus* counts were suggested to be among the effects of bacitracin (55 ppm) and oxytetracycline (2.5 ppm) that could explain the improvement of feed efficiency in broilers from days 0 to 21 (Lee et al., 2011).

### Growth promoters and resistance

The use of antibiotics in poultry production and the attendant selection of resistant bacteria has been the subject of numerous studies (Aarestrup, 2000; Angulo et al., 2000; O'Brien, 2002; Butaye et al., 2003; Asai et al., 2005; Anonymous, 2007; Castanon, 2007; Diarra et al., 2007; Diarrassouba et al., 2007). However, besides the simple principle that exposure to an antimicrobial agent can select for a resistant bacterium, the selection and dissemination of antimicrobial resistance is a complex phenomenon, which should be examined with ecological and population perspectives. Several studies have shown the presence of antibiotic resistant bacteria (*E. coli, Salmonella* serovars; *Enterococcus* spp., *C. perfringens*) in Canadian poultry (Diarrassouba et al., 2007; Diarra et al., 2010; Slavic et al., 2011; Agunos et al., 2012; St. Amand et al., 2013). Many antibiotic resistance genes in these bacteria have been identified on mobile genetic elements such as plasmids, transposons and integrons, allowing their dissemination among bacteria in the chicken gut or in extra-intestinal environments. However little is known about the selection, distribution and dissemination of antibiotic resistance genes in Canadian broiler chicken productions in relation to the use of specific therapeutic agents or antimicrobial growth promoters.

Recently, the Canadian Integrated Program for Antimicrobial Resistance Surveillance (CIPARS) reported a possible association between ceftiofur-resistant *Salmonella enterica* serovar Heidelberg isolated from retail chicken meats and the incidence of ceftiofur-resistant *Salmonella* Heidelberg infections in humans across Canada (Dutil et al., 2010). In the province of Quebec, the prophylactic use of ceftiofur in broiler chickens coincided with the rise of the prevalence of ceftiofur resistance in *Salmonella* that significantly decreased following voluntary withdrawal of this antibiotic (Rosengren et al., 2009). In relation to this, it is noteworthy to mention that the presence of β-lactam resistant *Salmonella enterica* serovars Kentucky, Typhimurium, Enteritidis, and Heidelberg that harbored a variety of important β-lactamase genes (CMY, TEM, SHV) either alone or in combination with other resistance genes were reported in chickens (Diarra et al., 2014). This observation is of concern because the use of cephalosporins at therapeutic levels can decrease the susceptibility to other antibiotics such as tetracycline and amikacin which resistance genes can be co-located on CMY-2 plasmids (Hamilton et al., 2012).

Using antimicrobial agents in feed, it was demonstrated that multi-antibiotic-resistant *E. coli* can colonize and persist in the broiler gut. Of 256 *E. coli* isolates analyzed using DNA-microarray, 88% possessed at least one antimicrobial resistance gene with 42% showing multiple resistance genes (Diarra et al., 2007). The bacterial phenotypes and distribution of resistance determinants in *E. coli* were found to be modulated by feed supplementation with some of the antimicrobial agents used in broiler chicken production (Diarra et al., 2007; Thibodaux et al., 2008; Bonnet et al., 2009). In *E. coli*, class 1 intregron and the aminoglycosides resistance *aadA* gene were predominantly found in the isolates from bacitracin and salinomycin treatments (Diarra et al., 2007), while the streptogramin resistance *vatD* gene was more prevalent in enterococci isolated from virginiamycin-treated birds compared to that found in the control birds (Thibodaux et al., 2008). Detailed antibiotic resistance genotypes of a variety of enterococci isolated from the feces and ceca of Canadian commercial broiler chickens were reported (Diarra et al., 2010). Genes conferring resistance to aminoglycosides (*aac, aacA-aphD, aadB, aphA, sat4*), macrolides (*ermA, ermB, ermAM, msrC*), tetracycline (*tetL, tetM, tetO*), streptogramins (*satG_vatE8*), bacitracin (*bcrR*), and lincosamide (*linB*) were detected in corresponding resistant *E. faecium* and *E. faecalis* strains (Diarra et al., 2010). Although food-producing animals are not considered as a source of *Enterococcus* infection in humans, antibiotic-resistant enterococci from these animals may transfer their resistance genes to bacterial strains infecting humans. Thus, the prevalence of antibiotic-resistant enterococci, in poultry can constitute a serious public health problem.

Accurate estimates of the volume of antimicrobials specifically used as growth promoters in Canadian animal productions including poultry is lacking. According to the Canadian Institute of Animal Health estimates reported par CIPARS (Government of Canada, 2013b), a total of 1,766,126, 1,617,747, 1,615,571, and 1,632,364 kg of antimicrobials were distributed in Canada for use in animals in 2006, 2007, 2008, and 2009, respectively. During these years, tetracyclines which are broad spectrum agents, ranked first with 48.0, 46.6, 42.1, and 42.1% of all antimicrobials being used in 2006, 2007, 2008, and 2009, respectively. The total amount of tetracyclines used specifically in poultry production is unknown. However, a high prevalence of tetracycline resistance in both Gram negative and Gram positive bacteria has been reported in Canadian poultry farms and poultry meats which could be related to the extensive used of this antibiotic.

In Gram negative bacteria such as *E. coli*, tetracycline resistance is frequently mediated by several efflux genes. The *tetB*, one of such genes, seems to be the most prevalent in *E. coli* isolated from Canadian broilers (Diarrassouba et al., 2007; Bonnet et al., 2009). The tetracycline resistance genes can be associated with large plasmids, which often carry other antibiotic resistance genes, heavy metal resistance genes, and/or other pathogenic factors such as toxins (Forgetta et al., 2012). Hence, selection for any of these factors selects for these plasmids. Associations between the β-lactamase (*tem*), tetracycline (*tet*), sulfonamide (*sulI* or *sulII*), aminoglycoside [*ant(3″)-Ia* (*aadA*)] and phenicol resistance (*floR*) genes and class 1 integrons were reported in *E. coli* isolated from broilers (Diarra et al., 2007). These associations increase the risk of selection and dissemination of resistance.

In Gram positive bacteria, the *tetL* gene encodes a large protein which confers resistance to tetracycline by active efflux while *tetM* encodes a cytoplasmic ribosome protecting protein also leading to resistance. The *tetL* and *tetM* genes were the most frequently found in association with the *ermB* gene (encoding resistance to macrolide, lincosamide and streptogramin B quinupristin-dalfopristin) and the bacitracin resistance gene *bcrA* in enterococci isolated from broiler chickens (Diarra et al., 2010). As mentioned above, bacitracin is one of the antimicrobial agents used as a growth promoter and to prevent necrotic enteritis (Table 1). The use of this antibiotic can co-select for resistance to other unrelated antibiotics as well, which demonstrates that the spread of antimicrobial resistance is a complex phenomenon.

The origin of the antimicrobial resistant bacteria colonizing the broiler gut needs to be established. In our laboratory, examination of the gut contents of day-old chicks revealed the presence of about 1.6 Log CFU of enterococci spp. per gram (Diarra, unpublished data). Some of these isolates were multi-resistant to bacitracin, ciprofloxacin, erythromycin, tylosin, flavomycin, streptomycin, kanamycin, lincomycin, quinupristin-dalfopristin, and tetracycline (Table 2). In Canada and other countries where poultry production is intensive, high numbers of broilers are raised in confined and non-sterile environments. Broilers can be exposed to such environmental bacteria among which some could be resistant. For example, chicken feed has been shown to contain *E. coli, Klebsiella* and *Pseudomonas* spp. isolates resistant to four to nine antibiotics (Saleha et al., 2009). In another study, examination of 23 commercial broiler feed samples and of 66 samples of raw feeding materials revealed that feedstuffs and poultry feed are extensively contaminated with resistant enterococci in agreement with our observations (Table 2) and, to a lesser extent, by *E. coli* (da Costa et al., 2007). Note that other factors also contribute to bacterial gut colonization such as the age of the animals and the microflora may thus vary over time. This should be taken into account when assessing antimicrobial resistance prevalence.

## Environmental perspectives

Poultry litter, a mixture of materials including bedding, feces and feathers, is a valuable soil amendment that is rich in nutrients and can improve soil physical, chemical, and biological properties for agricultural crops (Brye et al., 2004). Most of the antimicrobial agents administrated through feed or water are not fully absorbed in the chicken gut and up to 90% of the administered dose of some of the antimicrobials can be excreted in the feces. Residues of chicken feed additives such as bacitracin, chlortetracycline, monensin, narasin, nicarbazin, penicillin, salinomycin, and virginiamycin can be detected in the litter at concentrations ranging from 0.07 to 66 mg/L depending on the compounds (Furtula et al., 2010). Such a litter, if not treated to remove these compounds, may be an important source of antimicrobial residues when used as fertilizer. These residues also could contribute in the selection of antibiotic resistant bacteria as demonstrated for ceftiofur residues by Call et al. (2013).

Litter can be a source of antimicrobial resistant bacteria as well. Various antibiotic resistant *E. coli* strains harboring genes conferring resistance to β-lactams (*bla*_CMY−2_, *bla*_TEM_), tetracycline (*tetAB*) and streptomycin (*strAB*) have been reported to survive for several months in soil following late summer litter application (Merchant et al., 2012). Estimating survival of antibiotic resistant and potential pathogenic bacteria in soil amended with raw untreated litter from broiler fed antimicrobial supplemented diets is essential for developing intervention strategies against resistant pathogens and toward pathogen control in agricultural soils.

Drinking water should be very low in bacterial counts and no pathogenic microorganism should be detected in it. From 2005 to 2006, a bacteriologic study on 353,388 drinking water samples from private wells in Alberta and Ontario found that 4.6% of these samples were contaminated with *E. coli.* Antibiotic susceptibility tests done on 7063 of these *E. coli* isolates showed that 10.5% were resistant mainly to tetracycline, sulfonamides, β-lactams or aminoglycosides (Coleman et al., 2013). These authors reported that such antibiotic resistant *E. coli* were more commonly isolated from farms housing chickens or turkeys than from properties without poultry.

Primary biological aerosols (airborne biological particles derived from, or which are composed of living microorganisms) are of special concern in poultry barns and slaughterhouses, where the high number of chickens handled in these facilities leads to the presence of substantial concentrations of bacteria and other microorganisms in air (Donham et al., 2000). Antibiotic-resistant bacteria have been reported in broiler chicken air (Brooks et al., 2010; Vela et al., 2012). Biofilm forming staphylococci harboring genes conferring resistance to tetracycline (*tetK*), lincomycin (*linA*), erythromycin (*ermB*), and β-lactams (*blaZ*) were isolated from air inside and outside broiler production facilities (Vela et al., 2012). The airborne dispersion of antimicrobial resistant bacteria should not be underestimated since the presence of pathogenic bacteria in air represents a potential risk to poultry farm workers and to people working or living near these facilities.

## Concerns about food safety and spread of antibiotic resistance

Antibiotic-resistant bacteria constitute a major food safety issue. Antibiotic-resistant bacterial pathogens such as *Salmonella* or *E. coli* can infect humans through contact or consumption of contaminated food while non-pathogenic resistant isolates can transfer their resistant genes to human pathogens. Although multifactorial, practices contributing to the selection of antibiotic resistant bacteria include antibiotic use in livestock feed, and concerns about food safety and reduced efficacy of antibiotic treatment in human medicine have stimulated expert groups to action (Mathew et al., 2007; Laxminarayan et al., 2013).

Antibiotic resistance has become a worldwide threat to public health. For example in the United States of America (USA), according to a recent report from the Centers for Disease Control and Prevention (CDC), at least 2 million people become infected with “antibiotic resistant bacteria” among which at least 23,000 people die each year as a direct result of these infections (CDC, 2013). The USA National Antimicrobial Resistance Monitoring System (NARMS) assisted by the Food and Drug Administration (FDA) and the Department of Agriculture (USDA), monitor antimicrobial susceptibility of enteric bacteria from humans, retail meats and food-producing animals, in order to make decisions related to the approval of safe and effective antimicrobial drugs for animals (NARMS, 2012). In Canada, the Public Health Agency of Canada and the CIPARS track antimicrobial resistance to generate data helping to limit the spread of antibiotic resistant bacteria. More so, initiatives that collect data on commensal and environmental strains as reservoirs of antibiotic resistance genes are invaluable (Marshall and Levy, 2011). It is thought that the frequency of resistance genes in commensals may act as a marker of the emergence of resistance in pathogens (www.roarproject.org).

Concerns for safe food and effective medical antibiotics have pressured authorities for elimination of antibiotics as growth promoters as well as those of medical importance in animal production. Despite incomplete data, there were sufficient genuine and reasonable arguments for implementing such regulations in the European Union and similar policies and recommendations in North America were made based on the precautionary principle. For example, the CDC supports the strategy of the FDA to promote the judicious use of antibiotics that are important in treating humans. In Canada, there is a variety of efforts that follow this trend (Agunos et al., 2012). The Canadian Veterinary Medical Association is developing prudent and judicious antimicrobial use guidelines for veterinarians working with swine, beef or dairy herds and poultry flocks. The Veterinary Drugs Directorate (VDD) of Health Canada, which is responsible for the approval and registration of all antimicrobials for use in agriculture, is developing a risk management strategy to reduce the human health impact of antimicrobial resistance due to use of antimicrobials in animals.

Still, efficient control of foodborne pathogens remains a concern (Smadi and Sargeant, 2013) and removal of non-therapeutic antimicrobials from animal production may possibly increase the prevalence of pathogens in the animal gut and the frequency of foodborne illnesses. Alternatives to antibiotics are therefore required.

## Alternatives to antibiotics

Public pressure and concerns about food and environmental safety (antibiotic residues, spread of antibiotic genes and antibiotic-resistant pathogens) have driven researchers to actively look for alternative approaches that could eliminate or decrease the use of antibiotics while maintaining production yields and low mortality in poultry production. As discussed in previous sections, the biological basis for antibiotic effects on animal growth efficiency is most likely derived from effects on the intestinal microbiota, which in turn may reduce opportunistic subclinical infections, reduce the host response to the gut microflora, decrease competition for nutrients, and improve nutrient digestibility consequent to a reduction in some microbial fermentation by-products (Dibner and Richards, 2005). With such pleiotropic effects, it will be difficult to find alternatives to antimicrobials administered for prevention or provided as growth promoters in feed.

Several alternative strategies to antibiotics in poultry and livestock production are under investigation (Dahiya et al., 2006; Zakeri and Kashefi, 2011; Seal et al., 2013). Individual strategies examined included direct-fed microbial (probiotics) and live microbial feed supplements which beneficially affect the host animal by improving its intestinal balance (Rajput et al., 2013; Salim et al., 2013); prebiotics, indigestible feed ingredients that beneficially affect the host by selectively stimulating the activity of beneficial bacteria resident in the animal tract (Patterson and Burkholder, 2003; Baurhoo et al., 2009; Samanta et al., 2013); vaccination (Desin et al., 2013) and immune-stimulation through cationic peptides and cytokines (Asif et al., 2004; Kogut et al., 2013); bacteriocins and antimicrobial peptides (Joerger, 2003; Svetoch and Stern, 2010); bacteriophages (Huff et al., 2005, 2013; Zhang et al., 2013); organic acids with antimicrobial activities; herbs, spices and other plant extracts (González-Lamothe et al., 2009); and controlled organic productions with emphasis on diet formulation and ingredient selection, cereal type and dietary protein source and level (Drew et al., 2004; O'Bryan et al., 2008). To date, none of these strategies have been systematically implemented. Consequently, exploration for new approaches to prevent poultry diseases and colonization of poultry by foodborne pathogens is continuing worldwide.

### Berries as a generic source of bioactive molecules

Natural products as tools for disease prevention and health maintenance have reached public acceptance leading to an accelerated research in this area. There are now abundant reports of plant products with bioactivities against a wide variety of pathogenic bacteria. Multiple classes of antibacterial products, including phenolic acids and polyphenols, phenanthrenes, flavonoids, and terpenoids have been described and reviewed (González-Lamothe et al., 2009).

Some products may have antibacterial activities of their own by significantly altering growth or bacterial cell structures. Others which may be defined as “antibiotic potentiators or adjuvants” could allow reduction of antibiotic usage. Some may have anti-virulence effects or alter quorum-sensing necessary for efficient pathogenesis. Besides, others, defined as “immuno-stimulants” could assist the host immune system to adequately respond to the pathogen invasion, while others may positively affect the intestinal microbiota. Knowing that subclinical diseases caused by pathogens can impact productivity, this review presents some results on the potential of cranberry extracts to control pathogenic bacteria.

Cranberries, *Vaccinium macrocarpon* Aiton (Ericales: Ericaceae), are indigenous to wetlands of central and eastern North America (Eck, 1990). Canadian cranberry productions increased from 95,655 tons in 2009 to 134,575 tons in 2013. Most of the productions come from British Columbia, Quebec, New Brunswick, Nova Scotia and Prince Edward Island (Statistics Canada, 2013). Polyphenolic compounds are widely distributed in higher plants and are integral parts of the human diet. An important and often overlooked group of polyphenols is the proanthocyanidins (condensed tannins).

Particular interest is being shown in the proanthocyanidins from cranberry (Foo et al., 2000). Flavonoids in cranberry may reduce or prevent atherosclerosis by preventing oxidation of low density lipids (Reed, 2002). Cranberry proanthocyanidins at a concentration of 75 μ g/mL were found to inhibit the adherence of *E. coli* to urinary epithelial cells, preventing or mitigating thus UTI (Foo et al., 2000; Howell and Foxman, 2002). Cranberry extracts were also reported to inhibit the sialyllactose-specific adhesion of *Helicobacter pylori* to immobilized human mucus, erythrocytes, and cultured gastric epithelial cells (Burger et al., 2002). Because the inhibitors of adhesion are not necessarily bactericidal, the selection of resistant strains is unlikely to occur and anti-adhesion agents represent an interesting therapeutic strategy (Sharon and Ofek, 2002). The potential of plant tannins, including proanthocyanidins, as alternatives to growth promoters in poultry has recently been reviewed by Redondo et al. (2014). It is expected that the value of cranberry-based food and nutraceutical products will remain high as health benefits of cranberry become more firmly established. Recent studies suggest that the potential health effects of cranberry are associated with its phytochemical constituents (Blumberg et al., 2013). Furthermore, studies have revealed that extracts from these sources can affect various bacterial functions including disruption of their cell envelope, which parallels that of some antibiotics widely used as growth promoters in the poultry industry.

It has however been difficult to isolate specific active components from plant extracts which often consist of a mixture of a large number of structurally related compounds (Puupponen-Pimiä et al., 2005). These compounds have varying degrees of bioactivity or even opposing effects (growth inhibitors vs. growth stimulants) and even some with cytotoxicity (Jaki et al., 2008). Also, the spectrum of activity or the mode of action of purified components is often very narrow or non-specific and the use of berry extracts or pomace containing mixtures of bioactive compounds has become an attractive alternative to create an added value to berry by-products.

The antimicrobial activities of cranberry extracts were evaluated against important pathogenic Gram negative bacteria such as *E. coli* and *Salmonella enterica* serovar Typhimurium, which is often associated with poultry (Wu et al., 2008; Harmidy et al., 2011). It has been reported that treatment with cranberry proanthocyanidins (CPACs) inhibited *Salmonella* invasion and enteropathogenic *E. coli* pedestal formation, likely by perturbing the host cell cytoskeleton by CPACs rather than by an effect on bacterial virulence itself (Harmidy et al., 2011). Dehydrated, crushed cranberries or purified CPACs were also shown to inhibit the expression of the flagellin gene (*fliC*) in uropathogenic *E. coli* (Hidalgo et al., 2011).

In order to study the pleiotropic effects of cranberry extracts on *E. coli*, we (Gattuso et al., 2008) and others (Lin et al., 2011) have used a DNA array-based approach in an attempt to correlate specific transcriptional signatures with modes of action. The effects observed on the transcriptome of *E. coli* exposed to cranberry extracts correlated with known characteristics of cranberry constituents such as condensed tannins (flavonoids) and phenolic acids that could possibly act as iron chelators. In view of these results, cranberry extracts could be used to perturb bacterial iron homeostasis and improve nutritional immunity in the gut (Hood and Skaar, 2012).

**Figure 1:**
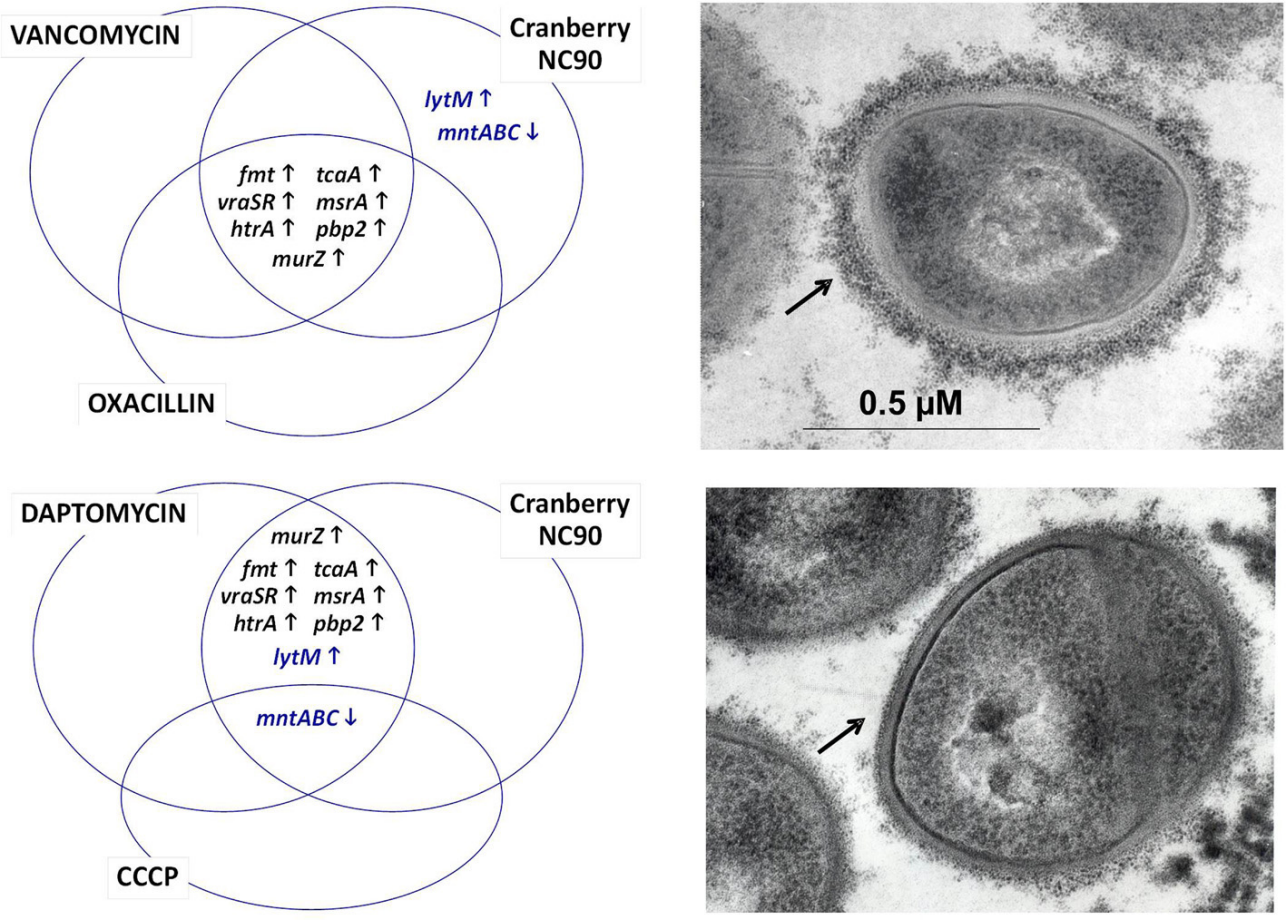
**Venn diagrams showing some of the *S. aureus* genes up- and down-regulated following exposure to cranberry (left)**. The transcriptional signature resembles to that of the cell wall stress stimulon provoked by peptidoglycan biosynthesis inhibitors such as vancomycin, oxacillin, and to some extent daptomycin. Compounds causing membrane depolarization like daptomycin and CCCP also share a common transcriptional signature with cranberry. Genes up- and down-regulated are represented by up and down arrows, respectively. Genes affected by cranberry also include those involved in capsular polysaccharide biosynthesis (Diarra et al., 2013), which correlates with the presence of a thinner capsule at the surface of *S. aureus* (lower right panel compared to the untreated control, top right panel). The capsule material (indicated by arrows) was labeled using polycationic ferritin as described before (Diarra et al., 2002).

Based on our own experience, commercially available cranberry products like Nutricran®90 (NC90) and some of our own cranberry extracts yielded stronger growth inhibition effects against Gram positive pathogens such as *Staphylococcus aureus* (Diarra et al., 2013), *Listeria monocytogenes* (Block et al., 2012), and *Clostridium perfringens* (Delaquis et al., 2010), although the minimal inhibitory concentrations of the cranberry products were several times higher than that of conventional antibiotics such as penicillin. Similarly to work done with *E. coli*, transcriptional analyses by microarrays allowed determining the modes of action of the cranberry product NC90 against *S. aureus* (Diarra et al., 2013). The effect of cranberry on the *S. aureus* transcriptome yielded the identification of several bacterial genes known to be up-regulated by the presence of cell-wall acting antibiotics, such as oxacillin, vancomycin, and daptomycin (Singh et al., 2001; Utaida et al., 2003; Muthaiyan et al., 2008), as represented in Figure 1. More specifically, a group of genes known as the cell wall stress regulon was strongly up-regulated and clearly demonstrated an effect of cranberry on *S. aureus* cell wall biosynthesis. Ethanol extraction of pomaces (pressed cakes) from fresh fruits also produced a cranberry fraction (FC111) modulating the same marker genes as demonstrated by qPCR. *S. aureus* cell surface disruption by cranberry is also supported by work from Wu et al. (2008) and by cell wall biosynthesis assays (Diarra et al., 2013). Besides, it was noted that NC90 and FC111 also modulated the expression of some *S. aureus* genes (like *lytM*, Figure 1) that respond to membrane depolarization, as provoked by carbonyl cyanide *m*-chlorophenylhydrazone (CCCP) and daptomycin (Muthaiyan et al., 2008). Interestingly, cranberry extracts also strongly down-regulated capsular biosynthesis genes, an effect that was corroborated by electron microscopy (Figure 1).

*Listeria* spp. are important foodborne pathogens that can be associated with various foods including fresh and frozen meat and poultry. Cranberry fraction FC111 also showed bactericidal effects as well as antibiofilm formation activities against *Listeria monocytogenes* (Block et al., 2012). Apostolidis et al. (2008) reported a proline dependent inhibition of *L. monocytogenes* by combinations of phenolic extracts of oregano and cranberry in both broth and cooked meat studies. These data indicate that further examination of the antimicrobial potential of cranberry extract is warranted (Wu et al., 2008).

The multiple biological effects of cranberry observed against *E. coli, Salmonella, S. aureus, C. perfringens*, and *Listeria*, certainly reflect the complexity of its composition and physical properties. The cranberry tannins include polyphenols and more specifically anthocyanins, flavonols and flavan-3-ols (Puupponen-Pimiä et al., 2005). Flavonoids, including anthocyanins and proanthocyanidins, are believed to be the major antimicrobial components (Puupponen-Pimiä et al., 2001). At this time, our mass spectrometry analysis of cranberry fraction FC111 could not determine if the observed antibacterial activity originates from iridoids, phenolics, or flavonoid components. Besides, we showed that the cranberry fraction FC111 obtained from pomace is an excellent natural polyphenolic product with potent antioxidant and vasorelaxant properties (Harrison et al., 2013), which combined with its antibacterial activities might represent an interesting alternative in poultry production. In this regard, a poultry feeding trial using a commercial whole cranberry fruit extract showed that a concentration of 40 mg of cranberry extracts per kg of feed induced low early mortality rates (improvement by 40% compared to the control) in birds. The mechanism of action leading to this improvement remains to be determined. However, diet supplementation with such extracts caused a shift of the intestinal tract bacterial population while not altering any broiler meat properties (Leusink et al., 2010).

### Cyclic diguanosine monophosphate (c-di-GMP)

c-di-GMP is a bacterial intracellular second messenger controlling diverse bacterial processes. This molecule is important for a wide range of pathogenic agents as it is involved in the modulation of the infection process through modulation of motility, cell adhesion and biofilm formation (Tamayo et al., 2007; Bordeleau et al., 2011). However, c-di-GMP is also a potent immuno-stimulatory agent that can modulate the host immune response and several reports demonstrated its adjuvant and therapeutic properties (Brouillette et al., 2005; Karaolis et al., 2007; Ogunniyi et al., 2008; Hu et al., 2009). Moreover, the ability of c-di-GMP as a mucosal adjuvant was also documented (Ebensen et al., 2007; Zhao et al., 2011). c-di-GMP might thus represent an interesting alternative to non-therapeutic antibiotics used in poultry production.

The infectious bursal disease virus (IBDV, Gumboro disease) is one of the major immuno-suppressive viruses affecting broilers. This virus is highly contagious and represents a major economic threat in poultry production worldwide (Bumstead et al., 1993). Since the effects of c-di-GMP on chicken immune responses had not yet been investigated, we evaluated the humoral immune response following oral administration or intramuscular injection of c-di-GMP in conjunction with the IBDV vaccine S-706 in broiler chickens (Fatima et al., 2011). Results indicated that c-di-GMP stimulated IgA production in serum and confirmed the potential of this molecule as a mucosal adjuvant.

As mentioned above, an enteric pathogen of particular concern in poultry is *C. perfringens* Type A, the causative agent of necrotic enteritis (Timbermont et al., 2011). Hence, in an effort to explore strategies to control *C. perfringens*, we investigated the potential of c-di-GMP in a broiler challenge model (Fatima et al., 2013). We found that c-di-GMP can modulate *C. perfringens* colonization in the host ceca with no noticeable effect on the microbiota and the commensal bacterial community of the intestine. It will be interesting to investigate in more details the value of c-di-GMP as an in-feed additive in poultry production.

## Conclusion

Antibiotics are important tools for the treatment of old and emerging infectious diseases. Their efficacy for this purpose should be preserved as it is now well documented that their abusive and inappropriate use in humans, livestock and poultry selects for antibiotic resistant bacteria, compromising thus their therapeutic efficacy. One of questionable practices in animal agriculture is the use of non-therapeutic antimicrobials for growth promotion. Even if this practice was determinant in the past, its advantage in current modern agriculture including poultry production needs to be re-evaluated because of the actual prevalence of antibiotic resistant bacteria in livestock and poultry and their products worldwide. The presence of multi-drug resistant commensal bacteria (*Escherichia* spp., *Enterococcus* spp.) and foodborne pathogens such as non-typhoid *Salmonella* associated with poultry are some of the examples among others. It is imperative to determine the exact sources and ecology of these resistant bacteria in order to develop strategies to stop their spread. It is also urgent to develop alternatives to antimicrobial growth promoters that will not compromise livestock and poultry health as well as the actual industry productivity. Canadian studies in this area identified some promising sources of alternatives to antibiotics which have been discussed here.

### Conflict of interest statement

The authors declare that the research was conducted in the absence of any commercial or financial relationships that could be construed as a potential conflict of interest.
